# The Use of Penalized Regression Analysis to Identify County-Level Demographic and Socioeconomic Variables Predictive of Increased COVID-19 Cumulative Case Rates in the State of Georgia

**DOI:** 10.3390/ijerph17218036

**Published:** 2020-10-31

**Authors:** Holly L. Richmond, Joana Tome, Haresh Rochani, Isaac Chun-Hai Fung, Gulzar H. Shah, Jessica S. Schwind

**Affiliations:** 1 Jiann-Ping Hsu College of Public Health, Department of Biostatistics, Epidemiology & Environmental Health Sciences, Georgia Southern University, Statesboro, GA 30458, USA; hr04848@georgiasouthern.edu (H.L.R.); jt20902@georgiasouthern.edu (J.T.); hrochani@georgiasouthern.edu (H.R.); cfung@georgiasouthern.edu (I.C.-H.F.); 2 Jiann-Ping Hsu College of Public Health, Department of Health Policy and Community Health, Georgia Southern University, Statesboro, GA 30458, USA; gshah@georgiasouthern.edu

**Keywords:** COVID-19, social determinants of health, county, race, demographics, socioeconomic, community health rankings

## Abstract

Systemic inequity concerning the social determinants of health has been known to affect morbidity and mortality for decades. Significant attention has focused on the individual-level demographic and co-morbid factors associated with rates and mortality of COVID-19. However, less attention has been given to the county-level social determinants of health that are the main drivers of health inequities. To identify the degree to which social determinants of health predict COVID-19 cumulative case rates at the county-level in Georgia, we performed a sequential, cross-sectional ecologic analysis using a diverse set of socioeconomic and demographic variables. Lasso regression was used to identify variables from collinear groups. Twelve variables correlated to cumulative case rates (for cases reported by 1 August 2020) with an adjusted *r* squared of 0.4525. As time progressed in the pandemic, correlation of demographic and socioeconomic factors to cumulative case rates increased, as did number of variables selected. Findings indicate the social determinants of health and demographic factors continue to predict case rates of COVID-19 at the county-level as the pandemic evolves. This research contributes to the growing body of evidence that health disparities continue to widen, disproportionality affecting vulnerable populations.

## 1. Introduction

Coronavirus disease 2019 (COVID-19), the disease caused by severe acute respiratory coronavirus 2 (SARS-CoV-2), was first detected in late 2019 in Wuhan, China [1]. Since its emergence, COVID-19 has spread globally, causing massive morbidity and mortality worldwide [2]. The World Health Organization declared COVID-19 a public health emergency of international concern on 30 January 2020, and subsequently labeled it a pandemic on 12 March 2020 [3]. To date, there have been more than 18 million confirmed infections worldwide with over 675,000 deaths attributed to COVID-19 [2].

Early in the pandemic, evidence indicated minority groups and those with lower socioeconomic position suffered disproportionately from COVID-19, both in the United States (US) and abroad [4,5]. Historically, these same groups experienced an inordinate burden of disease, both infectious and chronic, in non-pandemic, economically-stable times [6,7,8]. Data on COVID-19-related morbidity and mortality in the United States confirmed African American and minority populations were again disproportionately affected [9,10,11]. A recent county-level ecological study of the US found counties located in metropolitan areas (250,000–1 million people) with a higher percentage of African Americans were independently associated with increasing case counts in US counties in June 2020 [12]. Another county-level ecological study of seven states found counties with a higher percentage of non-institutionalized, disabled people, especially disabled Whites, showed a lower infection rate. However, the same group had a higher rate of COVID-19 related mortality [13]. Mounting research indicates the COVID-19 pandemic exacerbates the socioeconomic circumstances that give rise to health disparities in the US.

The social determinants of health refer to factors, other than healthcare or public health, associated with differences in health status or health outcomes that are driven by socioeconomic conditions. Determinants could include the lack of educational access, in addition to community, home, and work circumstances that diminish the potential for an individual to fully realize mental and physical health and wellbeing [14]. The social determinants of health are known to affect outcomes in chronic diseases such as diabetes [15], as well as acute and infectious diseases [16]. For decades, it’s been well understood that the social determinants of health reveal racial and ethnic inequities [17], manifested through injustices of systemic racism related to living conditions, access to healthy foods, healthy lifestyles, education, and other social and cultural resources [18].

These imbalances in the social determinants of health and the subsequent health outcomes can be seen not just along racial and demographic lines, but also geographically in the US with disparities in health between rural and urban areas [19]. African Americans, especially in the South, and other rural minorities, experience differences in health outcomes when compared to White counterparts [20]. The early spread of COVID-19 disproportionately affected those living in urban areas. However, there is growing concern that as the disease moves into rural populations, these communities have decreased capacity to handle large numbers of patients [21]. It was previously predicted rural populations would see an increasing rate of COVID-19 morbidity and mortality due to aging populations, unfavorable socioeconomic status, and a lack of resources in critical areas such as transportation, employment, healthcare capacity, public health infrastructure, and food security [22].

To understand the driving factors behind SARS-CoV-2 infection rates, it is essential to take into account the dynamic, evolving nature of the pandemic. By developing predictive models that evaluate county-level factors related to COVID-19 infections, we can adequately prepare and protect communities where high transmission rates are likely to occur. Therefore, the objective of this research was to build a predictive model for SARS-CoV-2 infection rates in the state of Georgia that included covariates, such as known demographic, socioeconomic, and health data at the county-level.

## 2. Materials and Methods

### 2.1. Study Population and Data Sources

To accomplish our research objective, a cross-sectional ecologic study was conducted at the county-level across Georgia. Our outcome of interest was cumulative incidence rates by county (cases/100,000 residents) for each of the 159 Georgia counties as publicly provided by the Georgia Department of Public Health (GA DPH) on their website [23]. Exposure data for independent variables was collected through the Robert Woods Johnson Foundation’s County Health Rankings (CHR) public website and their data sources [24]. A database was constructed of the county-level demographic, socioeconomic, physical environment, and health indicators and used to build the predictive model between these variables and COVID-19 infection rates in all counties across Georgia. Georgia consists of 159 counties, representing a wide range of sizes and demographics. County-level continuous variables relating to demographics, socioeconomic, physical environment, and health indicators were collected for all counties across Georgia. If data was missing from the CHR due to small numbers or uncertain reliability, the original data source for CHR was located and used when available. If information was unavailable from the original data source, those county statistics were estimated by the mean of the geographically surrounding counties, such as the food environmental index. For rare events where data was suppressed due to small numbers, such as infant mortality, they were estimated as zero.

### 2.2. Outcome of Interest

The COVID-19 case definition for the GA DPH was an individual with positive molecular testing for SARS-CoV-2. Cases recorded by the GA DPH were reported through electronic lab reporting and the state electronic notifiable disease surveillance system, as well as via calls or faxes from providers [23]. The continuous dependent variable in our study was the cumulative number of confirmed cases per 100,000 residents in a county, as publicly reported by the GA DPH on 1 August 2020. Data for cases per 100,000 residents was log-transformed for normality before analysis. Cases were excluded from our analysis if they did not have a known county of residence in Georgia at the time of case reporting.

### 2.3. Data Analysis

Descriptive statistics, including mean, median, and standard deviation for all variables were calculated. Variables where data could not be ascertained or accurately estimated were omitted from the model selection process. CHR rankings for access to care, such as primary care physician rate, dentist rate, and mental healthcare provider rate, were excluded from analysis due to the small geographic county size and the possibility of residents from adjoining counties sharing providers. Racial demographics were separated into minority or non-Hispanic White. Table S1 reports the included and excluded variables in our analysis and their level of inclusion or exclusion in the county health rankings.

**Figure 1:**
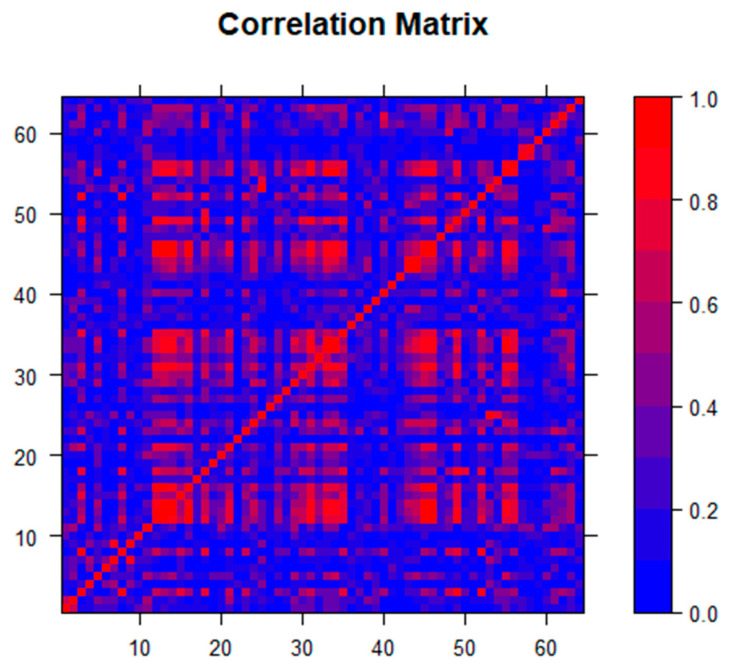
Correlation matrix for the 65 independent predictors used in the analysis, which shows multicollinearity and the need for lasso as the appropriate coefficient shrinkage technique.

Initial multivariable regression analysis revealed significant multicollinearity, which was not easily rectified using standard techniques related to variance inflation, condition index, and variance proportion diagnostics. A predictor collinearity matrix (Figure 1), created using the statistical programming language R v.3.6.3 (R Core Team, R Foundation for Statistical Computing, Vienna, Austria) revealed this multicollinearity, justifying the use of a technique other than the best subset multivariable regression analysis. Lasso (least absolute shrinkage and selection operator) regression analysis was used due to the number of predictors and overlap of variables. Figure 1 confirms that the selection of lasso for analysis was appropriate as opposed to other coefficient shrinkage techniques, such as elastic net [25]. By using coefficient shrinkage to zero, lasso variable selection allowed for the automated determination of the most important variables in a group of collinear determinants where traditional least-squares linear regression models failed, and variance of the least-squares estimators was unacceptably high. Proposed by Tibshirani in 1996 [26], lasso has been used in a variety of settings with similar sets of variables for outcomes with complex sets of underlying predictors [27,28,29]. Lasso’s prediction of variables was improved not only in settings where significant multicollinearity occurred but also when many predictors may be contributing small to moderate effects. Hence this technique is useful in the analysis of large data sets that include variables such as demographics, housing statistics, and economic indicators, which often overlap both within groups of variables as well as between groups. Lasso analysis was performed in SAS v9.4 (SAS Institute Inc. Cary, NC, USA) using the PROC GLMSELECT procedure and the default Schwarz Bayesian Information Criterion (SBC) for variable selection.

A sensitivity analysis was also performed by applying the same procedures to data from 1st April, 1st May, 1st June and 1st July, which provided multiple cross-sectional analyses to understand how predictive variables and overall predictive ability changed over time as the virus spread throughout the state.

## 3. Results

**Figure 2:**
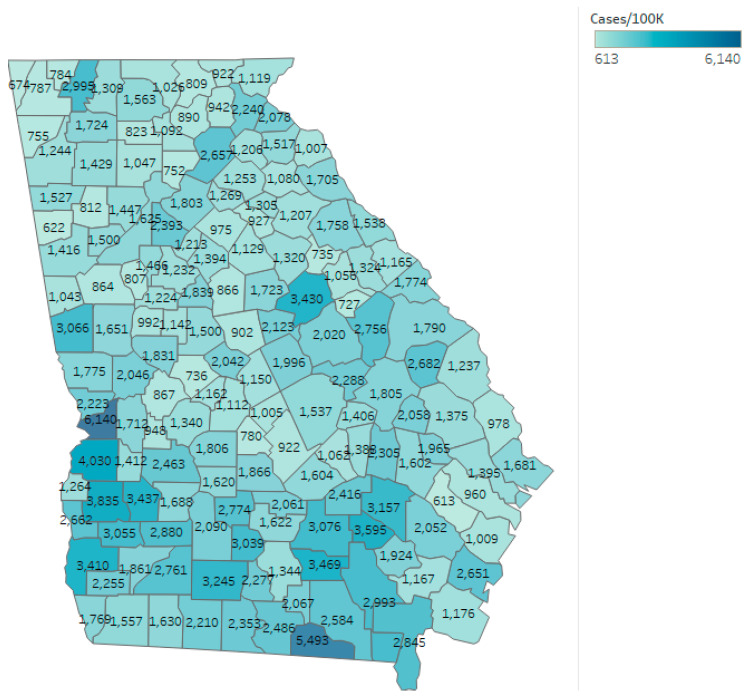
Cumulative COVID-19 cases per 100,000 for the state of Georgia as of 1 August 2020.

As of 1 August 2020, the confirmed cumulative rate reported by the DPH for the state of Georgia was 1726.62 per 100,000 residents, with 190,012 cumulative cases diagnosed in the state. The mean and median case rate for counties once non-residents and patients with unknown residency status were excluded was 1748.34 and 1538.46 per 100,000 residents, respectively. Cases per 100,000 residents varied from 612.60 in Long County to 6140.11 in Chattahoochee County. Long County reported 19,915 residents according to the DPH [23] and was 81% rural according to the CHR [25]. Chattahoochee County reported 10,749 residents and was 30% rural. The outcome of cumulative COVID-19 cases per 100,000 at the conclusion of the study are also shown by county in Figure 2.

Descriptive statistics, including mean, median, and standard deviation for case rates and the twelve variables chosen by lasso analysis on 1 August 2020, are shown in Table 1. Mean, median, and standard deviation for all independent variables considered are shown in Table S2. The overall Pearson’s correlation coefficient on 1 August 2020, was 0.4940 with an adjusted *r*-squared of 0.4525, indicating the final model had a moderate correlation with cumulative case rates by county. Lasso and other coefficient shrinkage methods eliminated some variables from analysis due to coefficient shrinkage to zero. Therefore, these standardized coefficients (*βz*) can be interpreted as they relate to each other within the model but should not be interpreted directly in terms of the dependent variable. Unlike traditional ordinary least squares regression, the coefficients do not directly represent a percent change in cumulative case rates in our model. Lasso analysis was used in our case to choose variables in the face of collinearity, as well as to identify variables that may only have a mild to moderate association.

Socioeconomic predictors in the final model included teen birth rate, children in poverty, children qualifying for free lunch, child mortality rate, and percentage of uninsured adults. The strongest indicators were those involving children, with the highest coefficient for the percent of children living in poverty (*βz* = 0.125). Additionally, children qualifying for free lunch (*βz* = 0.115), and child mortality rate (*βz* = 0.11) had a stronger positive association with increasing cumulative case rates relative to other variables in the final model. Lesser contributing variables were uninsured adults (*βz* = 0.078), and teen birth rate (*βz* = 0.035). Percent of non-Hispanic Whites (*βz* = –0.174) and percent of those with long commutes who drive alone (*βz* = −0.183) had the strongest standardized coefficients and were inversely related to cumulative case rates. In addition to minority status, other demographic indicators included were the percent of residents under 18 (*βz* = 0.034), percent of female residents (*βz* = −0.067), percent of residents not fluent in English (*βz* = 0.086) and Black/White segregation index (*βz* = 0.088). Other variables included were percent with annual influenza vaccine (*βz* = −0.062) and percent of those who self-report poor or fair health (*βz* = 0.09).

Our sensitivity analysis shown in Table 2 indicates how variables chosen by lasso analysis changed over time at monthly intervals, beginning 1 April 2020. Standardized coefficient (*βz*) estimates were included for each variable in the table. However, due to the mechanics of lasso analysis mentioned above, these should not be compared between models or in direct relation to cumulative case rates. We report them to show their negative or positive association with cumulative case rates, as well as to allow comparison within the models of the contribution of a variable to a model at a specific time point. Table S3 includes t statistics and *p*-values for each coefficient presented below.

A strengthening association of predictive variables with the outcome and a generally increasing number of chosen variables over time were observed. The adjusted *r*-squared on 1 April was 0.0930, with only one variable being predictive of cumulative case rates. By 1 August 2020, twelve variables were included in the model with an adjusted *r*-squared of 0.4540. On 1 April 2020, race was not predictive of higher cumulative case rates. However, by 1 May 2020, continuing until the final model on 1 August 2020, higher numbers of minorities were consistently predictive of counties with increased cumulative case rates. This variable was the most consistent variable included and was chosen in all models after 1 April. Some variables included in earlier time points were considered indicators of urban versus rural spread, such as higher levels of air pollution (PM_2.5_) and violent crime rates. With time, more indicators of socioeconomic status, such as low birthweight and lack of insurance, entered the model. The overall increase in adjusted *r*-squared and the number of socioeconomic variables predictive of increased case rates show that with the spread of COVID-19 over time in the state, the social determinants of health became increasingly predictive of higher cumulative case rates in the counties.

## 4. Discussion

Sequential lasso regression analysis showed an increasing trend of the predictive value of the social determinants of health on COVID−19 cumulative case rates. In our final analysis on 1 August 2020, our model using county-level demographics, health, access, and socioeconomic measures accounted for 45.4% of the variation in cumulative case rates by county. Additionally, we observed the number of variables included in the model by lasso regression, as well as model strength, increased as the pandemic progressed.

Our findings contribute to a growing body of literature that highlights the need to improve our understanding of the complex interconnectivity between demographics, socioeconomics, and structural inequities as they pertain to infectious diseases. Our study was also consistent with the finding of nationwide community-level disparities in COVID−19 infections and deaths in large US metropolitan areas [30]. Health disparities among race and ethnic divisions are not unique or specific to COVID−19, having been observed in a variety of infectious and chronic diseases [14,31,32]. Instead of proactively protecting those known to be the most vulnerable in society, the gaps in health disparities continue to widen during this crisis. These findings correspond with others that indicated minority groups were overrepresented in low-wage jobs considered essential, such as transportation and grocery store workers [33]. Additionally, another study found fewer than one in five Black Americans have job flexibility to work from home compared to more than a third of White and Asian American workers [34]. Thus, racial differences seen in our study and others may be related to a variety of reasons, including a varying ability to social distance and differences in access and quality of care [35], as well as differences in perceived susceptibility to infectious diseases [36]. Our research supports these explanations, as the inverse association between non-Hispanic Whites and cumulative case rates was the most consistent variable included over time and had one of the strongest coefficients in our final model, alongside a variety of other demographic and socioeconomic indicators.

The negative association of percent of residents with long, solo commutes in the model was first seen in the analysis on 1 July and was the most influential variable in the final analysis on 1st August. In general, this variable is considered indicative of poor health and chronic diseases, such as obesity, diabetes, hypertension, and cardiopulmonary disease [37,38,39]. We suspect this addition represented residents of suburban communities, who may be telecommuting during the crisis. Wealthy suburban residents may be more likely to have occupations that allow working from home, as opposed to urban residents, and may be additionally advantaged due to low crowding and a higher possibility of social distancing. This association between the ability to work from home and socioeconomic status has been previously reported [40]. Research elsewhere supports such variation in COVID-19 cases by geolocation [41].

Although it was a small contributor in terms of its impact to the final model (*βz* = 0.034), the addition of percent of people under 18 years old as a variable in the August 1 model is worth discussion. This variable was not seen previously in our sensitivity analysis. Its addition may be an aberration, but in light of concern over younger individuals becoming infected and spreading the infection [42], this association could also become stronger with time. There is a known increased risk of morbidity and mortality for older adults and seeming resistance to severe disease outcomes by young adults and children who may nonetheless be spreading the virus [43]. The increased availability of testing may play a part in the inclusion of this variable, as children, teens, and their young parents may now test at a higher rate even if they do not present with severe symptoms. Additionally, as an ecologic study, the inclusion of this variable could indicate an infection of adults or parents with children under the age of 18 in the household rather than the children themselves. Further research and monitoring are needed as children return to school.

Our study has several important limitations that should be taken into account. First, most of the county-level variables used as independent variables were measured by a variety of organizations across a two- to three-year time span for a purpose beyond COVID-19. Second, the implementation of new state policies for the mitigation of COVID-19, including stay-at-home orders, social distancing, and mask ordinances, may have impacts not measured through this cross-sectional research. Furthermore, our unit of analysis was the county. Therefore, aggregation bias should be considered as the relationships observed on the county-level may not hold up on the individual level. Our methodological rigor in the selection of covariates for the final model through lasso regression may also be a limitation as opposed to selecting the independent variables based purely on theoretical reasoning. Lasso regression analysis has been shown to over-select regression coefficients, which is a concern and drawback for this method. However, it still was shown to be superior to ordinary least squares techniques in similar situations [44,45].

Since COVID-19 is caused by a novel coronavirus, we believe validating traditional epidemiological techniques using computer learning models, such as lasso, can add support to previous findings related to race and have the additional ability of identifying variables that contribute small or moderate effects to COVID-19 infection rates. Additional research is needed to further explore the complicated relationship between COVID-19 pathogenesis, environmental factors, demographics, and socioeconomics with regard to the social determinants of health. We hope the use of the lasso in this study serves as another methodology that can be used to investigate other outcomes of COVID-19 and their relationship to the social determinants of health, such as cause-specific mortality and hospitalization rates. Due to surveillance gaps in this rapidly spreading disease, there have been challenges in collecting and obtaining individual-level information that can help address the concerns with an ecologic study. Combining individual-level data with neighborhood effects through the use of multilevel modeling could provide a clearer picture of factors related to COVID-19 diagnoses and mortality. Finally, since this is an ecologic, cross-sectional examination of COVID-19 in the state of Georgia, causal inference should not be extrapolated from these findings. However, our final model and sensitivity analysis provide a great starting point for future longitudinal research. The consistency of our findings with the disparities and inequalities observed across the country in morbidity and mortality rates suggest many structural-level issues are contributing to the spread of COVID-19 [46].

## 5. Conclusions

This research examined the community-level impact of factors from both a health and economic perspective on county-level COVID-19 case rates in the state of Georgia. Because health, demographic, and socioeconomic factors overlap in very complex ways, the full scale and intricacy of these inter-linkages are difficult to ascertain. However, we believe the strategic use of computer learning techniques, such as lasso, can elucidate some of these complexities. In the absence of consistent data collection on the demographics of positive cases, group-level studies such as ours help to identify influential predictors. Given the knowledge that the social determinants of health have significant effects on acute and chronic disease burden within a population, these findings support the linkage between fragile health, economic indicators, and demographics as key predictors of infection rates. Until longstanding inequities are eliminated and systemic injustices are addressed, the health and wellbeing of vulnerable and minority populations across Georgia will continue to be disproportionately affected, leaving marginalized communities to shoulder the largest burden of COVID-19.

## Figures and Tables

**Table 1:** Descriptive statistics for case rates and all independent variables chosen by lasso analysis.

Variable	Median	Mean	Standard Deviation	Standardized Coefficient *βz*
Cumulative cases/100,000 residents	1538.46	1748.341	889.452	
Log transformed case rates (used for analysis)	7.3385	7.3563	0.4629	
Percent with long commute who drive alone	36.8	36.92	11.95	−0.1828
Percent non-Hispanic White residents	61.78	61.96	17.26	−0.1741
Percent of children qualifying for free lunch	74.61	73.43	20.47	0.1154
Percent who report poor or fair health	20.16	20.1	3.73	0.0897
Percent not proficient in English	0.93	1.62	1.95	0.0856
Segregation index: Black/White	29.9	29.52	15.09	0.0876
Percent of uninsured adults	19.74	20.1	3.27	0.0779
Percent female	51.11	50.39	3.2	–0.0671
Percent with annual influenza vaccine	42	41.13	5.62	–0.0622
Teen birth rate	37.23	35.79	13.4	0.0351
Percent under 18 years of age	22.78	22.4	3.14	0.0344
Child mortality rate	71.96	73.1	24.26	0.0114

**Table 2:** Variables chosen by lasso analysis across time points of the COVID-19 pandemic.

Category	4/1/2020	5/1/2020	6/1/2020	7/1/2020	8/1/2020
**Demographics**		Percent non-Hispanic White (*βz* = −0.056)	Percent non-Hispanic White (*βz* = −0.11)	Percent non-Hispanic White (*βz* = −0.055)	Percent non-Hispanic White (*βz* = −0.174)
					Segregation index—Black:White (*βz* = 0.088)
					Percent under 18 years of age (*βz* = 0.034)
					Percent female (*βz* = −0.067)
					Percent not proficient in English (*βz* = 0.086)
**Health indicators**				Percent who report poor or fair health (*βz* = 0.192)	Percent who report poor or fair health (*βz* = 0.09)
**Access to care**		Women with annual mammography (*βz* = 0.055)			
					Percent with annual flu vaccine (*βz* = −0.062)
**Urban vs. rural spread**		Violent crimes rate (*βz* = 0.14)	Violent crimes rate (*βz* = 0.063)		
				Long commute who drive alone (*βz* = −0.066)	Long commute who drive alone (*βz* = −0.183)
		Average daily PM_2.5_ (*βz* = 0.069)			
**Socioeconomic**	Children in single-family homes (*βz* = 0.132)	Children in single-family homes (*βz* = 0.012)			Teen birth rate (*βz* = 0.035)
			Children in poverty (*βz* = 0.116)		Children in poverty (*βz* = 0.125)
			Low birthweight (*βz* = 0.015)	Low birthweight (*βz* = −0.076)	Children qualifying for free lunch (*βz* = 0.115)
		Child mortality rate (*βz* = 0.19)	Child mortality rate (*βz* = 0.042)		Child mortality rate (*βz* = 0.11)
				Uninsured adults (*βz* = −0.054)	Uninsured adults (*βz* = 0.078)
**Model adjusted *r*-squared**	0.0930	0.2011	0.1421	0.2322	0.4525
**Model F statistic**	16.99	7.63	6.23	10.56	11.88
**Model *p*-value**	<0.0001	<0.0001	<0.0001	<0.0001	<0.0001

## Supplementary Materials

The following are available online at https://www.mdpi.com/1660-4601/17/21/8036/s1. Table S1: Variables included and excluded from our database and their level of inclusion in the County Health Reports (CHR) calculations. Table S2: Descriptive statistics for all variables included in the research. Table S3: t statistics and associated *p*-values for all variables included in analysis.

## References

[1] Li Q., Guan X., Wu P., Wang X., Zhou L., Tong Y., Ren R., Leung K.S.M., Lau E.H.Y., Wong J.Y. (2020). Early transmission dynamics in Wuhan, China, of novel coronavirus–infected pneumonia. N. Engl. J. Med..

[2] Johns Hopkins University COVID-19 Case Tracker. https://coronavirus.jhu.edu.

[3] Ghebreyesus T. WHO Director-General’s Opening Remarks at the Media Briefing on COVID-19. https://www.who.int/dg/speeches/detail/who-director-general-s-opening-remarks-at-the-media-briefing-on-covid-19---11-march-2020.

[4] Mahajan U.V., Larkins-Pettigrew M. (2020). Racial demographics and COVID-19 confirmed cases and deaths: A correlational analysis of 2886 US counties. J. Public Health.

[5] Abuelgasim E., Saw L.J., Shirke M., Zeinah M., Harky A. (2020). COVID-19: Unique public health issues facing Black, Asian and minority ethnic communities. Curr. Probl. Cardiol..

[6] Gao S., Jiang F., Jin W., Shi Y., Yang L., Xia Y., Jia L., Wang B., Lin H., Cai Y. (2020). Risk factors influencing the prognosis of elderly patients infected with COVID-19: A clinical retrospective study in Wuhan, China. Aging.

[7] Bambra C. (2016). Health Divides-Where You Live Can Kill You.

[8] Hatch S.L., Frissa S., Verdecchia M., Stewart R., Fear N.T., Reichenberg A., Morgan C., Kankulu B., Clark J., Gazard B. (2011). Identifying socio-demographic and socioeconomic determinants of health inequalities in a diverse London community: The South East London Community Health (SELCoH) study. BMC Public Health.

[9] Garg S., Kim L., Whitaker M., O’Halloran A., Cummings C., Holstein R., Prill M., Chai S.J., Kirley P.D., Alden N.B. (2020). Hospitalization rates and characteristics of patients hospitalized with laboratory-confirmed Coronavirus disease 2019-COVID-NET, 14 States, March 1-30, 2020. Mmwr. Morb. Mortal. Wkly. Rep..

[10] Stafford K., Hoyer M., Morrison A. (2020). Outcry Over Racial Data Grows as Virus Slams Black Americans. Associated Press News.

[11] Vahidy F.S., Nicolas J.C., Meeks J.R., Khan O., Jones S.L., Masud F., Sostman H.D., Phillips R.A., Andrieni J.D., Kash B.A. (2020). Racial and ethnic disparities in SARS-CoV-2 pandemic: Analysis of a COVID-19 observational registry for a diverse U.S. metropolitan population. medRxiv.

[12] Mourad A., Turner N.A., Baker A.W., Okeke N.L., Narayanasamy S., Rolfe R., Engemann J.J., Cox G.M., Stout J.E. (2020). Social disadvantage, politics, and SARS-CoV-2 trends: A county-level analysis of United States data. medRxiv.

[13] Olulana O., Abedi V., Avula V., Chaudhary D., Khan A., Shahjouei S., Li J., Zand R. (2020). Regional Association of Disability and SARS-CoV-2 Infection in 369 Counties of the United States. Medrxiv Prepr. Serv. Health Sci..

[14] Marmot M., Friel S., Bell R., Houweling T.A.J., Taylor S. (2008). Closing the gap in a generation: Health equity through action on the social determinants of health. Lancet.

[15] Walker R.J., Strom Williams J., Egede L.E. (2016). Influence of race, ethnicity and social Determinants of Health on Diabetes Outcomes. Am. J. Med Sci..

[16] Coffey P.M., Ralph A.P., Krause V.L. (2018). The role of social determinants of health in the risk and prevention of group A streptococcal infection, acute rheumatic fever and rheumatic heart disease: A systematic review. PLoS Negl. Trop. Dis..

[17] Lillie-Blanton M., Laveist T. (1996). Race/ethnicity, the social environment, and health. Soc. Sci. Med..

[18] Nazroo J.Y. (2003). The structuring of ethnic inequalities in health: Economic position, racial discrimination, and racism. Am. J. Public Health.

[19] Thomas T.L., DiClemente R., Snell S. (2013). Overcoming the triad of rural health disparities: How local culture, lack of economic opportunity, and geographic location instigate health disparities. Health Educ. J..

[20] Scott A.J., Wilson R.F. (2011). Social determinants of health among African Americans in a rural community in the Deep South: An ecological exploration. Rural Remote Health.

[21] Paul R., Arif A.A., Adeyemi O., Ghosh S., Han D. (2020). Progression of COVID-19 From Urban to Rural Areas in the United States: A Spatiotemporal Analysis of Prevalence Rates. J. Rural Health.

[22] Shah G.H., Shankar P., Schwind J.S., Sittaramane V. (2020). The detrimental impact of the COVID-19 crisis on health equity and social determinants of health. J. Public Health Manag. Pr..

[23] Georgia Department of Public Health COVID-19 Daily Status Report. https://dph.georgia.gov/covid-19-daily-status-report.

[24] University of Wisconsin Public Health Institute and Robert Wood Johnson Foundation County Health Rankings. https://www.countyhealthrankings.org/.

[25] Lu M., Zhou J., Naylor C., Kirkpatrick B.D., Haque R., Petri W.A., Ma J.Z. (2017). Application of penalized linear regression methods to the selection of environmental enteropathy biomarkers. Biomark. Res..

[26] Tibshirani R. (1996). Regression shrinkage and selection via the lasso. J. R. Stat. Soc. Ser. B (Methodol.).

[27] Simeonov K.P., Himmelstein D.S. (2015). Lung cancer incidence decreases with elevation: Evidence for oxygen as an inhaled carcinogen. PeerJ.

[28] Ortega Hinojosa A.M., Davies M.M., Jarjour S., Burnett R.T., Mann J.K., Hughes E., Balmes J.R., Turner M.C., Jerrett M. (2014). Developing small-area predictions for smoking and obesity prevalence in the United States for use in Environmental Public Health Tracking. Environ. Res..

[29] Park S.K., Mukherjee B., Xia X., Sparrow D., Weisskopf M.G., Nie H., Hu H. (2009). Bone lead level prediction models and their application to examine the relationship of lead exposure and hypertension in the Third National Health and Nutrition Examination Survey. J. Occup. Environ. Med..

[30] Adhikari S., Pantaleo N.P., Feldman J.M., Ogedegbe O., Thorpe L., Troxel A.B. (2020). Assessment of Community-Level Disparities in Coronavirus Disease 2019 (COVID-19) Infections and Deaths in Large US Metropolitan Areas. JAMA Netw. Open.

[31] David R., Messer L. (2011). Reducing Disparities: Race, Class and the Social Determinants of Health. Matern. Child Health J..

[32] Braveman P., Gottlieb L. (2014). The social determinants of health: It’s time to consider the causes of the causes. Public Health Rep..

[33] Cubrich M. (2020). On the frontlines: Protecting low-wage workers during COVID-19. Psychol. Trauma.

[34] Gould E., Shierholz H. (2020). Not Everybody Can Work from Home: Black and Hispanic Workers are Much Less Likely to be Able to Telework.

[35] Hargraves J.L., Hadley J. (2003). The contribution of insurance coverage and community resources to reducing racial/ethnic disparities in access to care. Health Serv. Res..

[36] Chen J.Y., Fox S.A., Cantrell C.H., Stockdale S.E., Kagawa-Singer M. (2007). Health disparities and prevention: Racial/ethnic barriers to flu vaccinations. J. Community Health.

[37] Gordon-Larsen P., Boone-Heinonen J., Sidney S., Sternfeld B., Jacobs D.R., Lewis C.E. (2009). Active commuting and cardiovascular disease risk: The CARDIA Study. Arch. Intern. Med..

[38] Cunningham S.A., Patel S.A., Beckles G.L., Geiss L.S., Mehta N., Xie H., Imperatore G. (2018). County-level contextual factors associated with diabetes incidence in the United States. Ann. Epidemiol..

[39] King D.M., Jacobson S.H. (2017). What Is Driving Obesity? A Review on the connections between obesity and motorized transportation. Curr. Obes. Rep..

[40] Baker M.G. (2020). Who cannot work from home? Characterizing occupations facing increased risk during the COVID-19 pandemic using 2018 BLS data. medRxiv.

[41] Reichberg S.B., Mitra P.P., Haghamad A., Ramrattan G., Crawford J.M., Berry G.J., Davidson K.W., Drach A., Duong S., Juretschko S. (2020). Rapid emergence of SARS-CoV-2 in the greater New York Metropolitan Area: Geolocation, demographics, positivity rates, and hospitalization for 46,793 persons tested by Northwell Health. Clin. Infect. Dis..

[42] Kelvin A.A., Halperin S. (2020). COVID-19 in children: The link in the transmission chain. Lancet Infect. Dis..

[43] Song J., Hu W., Yu Y., Shen X., Wang Y., Yan J., Yang X., Gong S., Wang M. (2020). A Comparison of clinical characteristics and outcomes in elderly and younger patients with COVID-19. Med. Sci. Monit..

[44] Antonacci Y., Astolfi L., Nollo G., Faes L. (2020). Information transfer in linear multivariate processes assessed through penalized regression techniques: Validation and application to physiological networks. Entropy.

[45] Haufe S., Müller K.-R., Nolte G., Krämer N. Sparse causal discovery in multivariate time series. Proceedings of Causality: Objectives and Assessment at MPIS 2008.

[46] Yancy C.W. (2020). COVID-19 and African Americans. JAMA.

