# Supplementary material for: The Use of Penalized Regression Analysis to Identify County-Level Demographic and Socioeconomic Variables Predictive of Increased COVID-19 Cumulative Case Rates in the State of Georgia
Source: Int J Environ Res Public Health. 2020 Oct 31;17(21):8036. doi: 10.3390/ijerph17218036 (PMC7663274; doi:10.3390/ijerph17218036)
Supplement: Supplementary file 1 [file ijerph-17-08036-s001.pdf]

International Journal of
Environmental Research and Public Health

Table S1. Variables included and excluded from our database and their level of inclusion in the County Health Reports (CHR) calculations.

Category	Included CHR Measures	Included Additional Measures	Excluded CHR Measures	Excluded Additional Measures
Health Outcomes		Premature Death	Life Expectancy	
		Premature Age Adjusted Mortality		
		Child Mortality		
		Infant Mortality		
Quality of Life	Poor or Fair Health Days	Frequent Physical Distress		
	Poor Physical Health Days	Frequent Mental Distress		
	Poor Mental Health Days	Diabetes Prevalence		
	Low Birthweight	HIV Prevalence		
Tobacco Use	Adult Smoking			
Diet and Exercise	Adult Obesity	Food Insecurity		
	Food Environment index	Limited Access to Health Food		
	Physical Inactivity			
	Access to Exercise			
Alcohol and Drug use	Excessive Drinking			
	Alcohol Impaired Drinking			Drug Overdose Deaths
Sexual Activity	STDs/Chlamydia Rate			
	Teen Birth Rate			
Other Behavior		MV Crash Deaths		
		Insufficient Sleep		
Access	Uninsured	Uninsured Adults		
		Uninsured Children	Primary Care Physicians	
	Preventable Hospital Stays		Dentists	
			Mental Health Providers	Other Primary Care Providers
Quality of Care	Annual Mammography			
	Flu Vaccine			
Education	High School Graduation			
	Some College			Reading Scores
Employment	Unemployment			Math Scores
Income	Children in Poverty	20th Income Percentile		Disconnected Youth
	Income Ratio	80th Income Percentile		Residential Segregation Black:White
	Children Living in Single Parent Homes	Median Household Income		Residential Segregation Non-White:White
	Social Associations Rate	Children Eligible for Free Lunch		
	Violent Crime	Homicides		
	Injury Deaths	Firearms Fatalities		Suicides
Physical Environment	Air Particulate Matter	Traffic Volume	Drinking Water Violations	Juvenile Arrests
	Severe Housing Problems	Homeownership		
	Driving to work	Severe Cost of Housing Burden		

	Long commute - driving alone	
Demographic	% 18 or Below	
	% 65 or Older	
	% Non-Hispanic White	% Non-Hispanic Black
	% Not Proficient in English	% American Indian & Alaskan Native
	% Females	% Asian
	% Rural	% Native Hawaiian/Other Pacific Islander

Table S2. Descriptive statistics for all variables included in the research.

Variables	Mean	Median	Standard Deviation	Variable	Mean	Median	Standard Deviation
Cases per 100,000	1748.34	1538.46	889.45	Income ratio	4.97	4.89	0.94
Ln(cases/100,000)	7.36	7.34	0.46	20th percentile income	18946.21	16756.00	6596.98
% less than 18 years of age	22.40	22.78	3.14	80th percentile income	90086.91	84685.00	20825.62
% 65 and over	17.52	17.13	4.55	% children in single parent homes	40.77	40.53	12.40
% Non-Hispanic White	61.96	61.78	17.26	social associations rate	8.98	9.05	3.47
% Not Proficient in English	1.62	0.93	1.95	Violent Crimes rate	307.70	273.88	219.74
% Female	50.39	51.11	3.20	Injury death rate	77.49	77.48	18.41
% Rural	60.49	64.71	28.97	Average daily PM2.5	10.74	10.90	0.62
% poor of fair health days	20.10	20.16	3.73	% severe housing problems	15.97	15.70	3.26
number of poor physical health days	3.85	3.88	0.44	% drive to work alone to work	82.41	82.59	5.40
number poor mental health days	4.07	4.10	0.30	% long commute drive alone	36.92	36.80	11.96
% low birthweight	10.01	9.82	2.04	Life expectancy	76.16	75.95	2.30
% smokers	18.26	18.25	2.30	Age adjusted death rate	466.91	472.82	93.50
% obesity	34.45	34.70	5.89	% frequent physical distress	12.13	12.19	1.61
Food environment index	7.08	7.20	1.22	% frequent mental distress	13.21	13.28	1.28
% physical activity	30.85	30.60	5.45	% adults with diabetes	14.67	14.00	4.84
% with access to exercise	54.17	59.30	26.79	HIV Prevalence Rate	311.88	264.50	224.51
% excessive drinking	14.90	14.73	1.92	% food insecurity	15.71	16.20	4.36
% Driving Deaths with Alcohol Involvement	22.69	22.22	11.61	% limited access to healthy foods	7.64	5.78	7.73
Chlamydia Rate	512.03	489.40	247.98	Motor Vehicle mortality rate	18.51	18.47	8.63
Teen Birth Rate	35.79	37.23	13.40	% insufficient sleep	37.04	37.12	2.69
% uninsured	16.54	16.30	2.62	% uninsured adults	20.10	19.74	3.27
% with annual mammography	40.01	40.00	5.16	% uninsured children	7.68	7.31	1.56
% with flu vaccine	41.13	42.00	5.62	Median household income	47507.08	43439.00	13626.51
% High School graduation	87.69	88.14	5.52	% free lunch	73.32	74.61	20.47
% some college	50.72	49.66	11.96	Segregation index B/W	29.53	29.91	15.09
% unemployment	4.44	4.25	0.92	Segregation index NW/W	26.30	26.04	12.84
% children in poverty	28.63	30.10	9.34	Firearms fatality rate	14.76	15.73	6.71
% severe housing cost burden	13.58	13.25	3.32	Traffic volume	96.23	49.79	173.63
Infant Mortality Rate	7.35	7.63	3.34	% homeownership	67.97	68.36	8.95
Child Mortality Rate	73.10	71.96	24.26				

Table S3. t statistics and associated *p*-values for all variables included in analysis.

Variable	Student's t test for μ0 = 0	*p*-value for t test
Percent non-Hispanic white	45.2597	<0.0001
Segregation Index - Black:White	24.6791	<0.0001
Percent under 18 years of age	89.9538	<0.0001
Percent female	198.6648	<0.0001
Percent not proficient in English	10.4926	<0.0001
Percent who report poor or fair health	67.9243	<0.0001
Women with annual mammography	97.7386	<0.0001
Percent with annual flu vaccine	92.2813	<0.0001
Violent crimes rate	17.6568	<0.0001
Long commute who drive alone	38.9434	<0.0001
Average daily $PM_{2.5}$	217.9835	<0.0001
Children in single-family homes	41.4615	<0.0001
Teen birth rate	61.9721	<0.0001
Children in poverty	38.6565	<0.0001
Low birthweight	61.9721	<0.0001
Children qualifying for free lunch	45.1642	<0.0001
Child mortality rate	37.9985	<0.0001
Uninsured adults	77.4152	<0.0001
